# Spontaneous functional full recovery from motor and sensory deficits in adult mice after mild spinal cord injury

**DOI:** 10.1016/j.heliyon.2019.e01847

**Published:** 2019-06-02

**Authors:** Yohei Kakuta, Anna Adachi, Marino Yokohama, Toshiki Horii, Tokue Mieda, Yoichi Iizuka, Kenji Takagishi, Hirotaka Chikuda, Haku Iizuka, Kazuhiro Nakamura

**Affiliations:** a Department of Orthopedic Surgery, Gunma University Graduate School of Medicine, 3-39-22, Showa-machi, Maebashi, Gunma, 371-8511, Japan; b Department of Laboratory Sciences, Gunma University Graduate School of Health Sciences, 3-39-22, Showa-machi, Maebashi, Gunma, 371-8511, Japan; c Department of Orthopedic Surgery, Saint-Pierre Hospital, 786-7, Kamisano-machi, Takasaki, Gunma, 370-0857, Japan

**Keywords:** Neuroscience

## Abstract

The extent of spontaneous recovery in patients with a spinal cord injury (SCI) has not been thoroughly investigated. It is essentially not known whether SCI animals exhibit full recovery from both motor and sensory deficits as well. Here, we developed an appropriate condition to produce a mild SCI in mice. Mice given a mild contusion SCI showed transient low performances in the Basso Mouse Scale for locomotion (BMS), rotarod and beam walking tests after the SCI, which was followed by complete restoration in a short time. The SCI mice also showed functional full recovery from low sensitivity to light touch using dynamic touch test. Nevertheless, the fully-recovered SCI mice still exhibited significant loss of myelin in the spinal cord. These results suggest a high potential of adaptation of motor and sensory systems in mice and might provide insight into the prognoses of SCI patients.

## 1 Introduction

Spinal cord injury (SCI) is one of the major traumas worldwide because SCIs can be caused by common traumas such as motor vehicle accidents, sports and falls in mountains as well as tumors and infections [1]. The signs and symptoms of an SCI include paralysis, bladder and bowel control failures and sexual dysfunction [2]. Neuronal damages in an adult caused by an SCI persist throughout life [3]. Since the majority of patients are young people, they suffer from the symptoms for quite a long time.

Motor performances are partially recovered spontaneously several weeks after an SCI in adult rodents. The Basso Mouse Scale for locomotion (BMS) [4], rotarod test [5, 6] and beam walking test [7] have been frequently used to evaluate motor functions after SCI in mice. In each behavioral test, the motor performances are worst immediately after the SCI. Then, the performances improve over several weeks thereafter. However, it is not essentially known whether the SCI mice show full recovery from sensory deficits as well.

Likewise, temporal correlations between the spontaneous regeneration of the spinal cord and the behavioral recovery in multiple tests after SCI has not been thoroughly studied. Mice at 42 days post-SCI with low BMS scores displayed extensive glial scar formation [8]. These data indicate that mice with low BMS scores demonstrate anatomical abnormalities at 42 days post-SCI. Conversely, it is not essentially known whether mice have a potential to show maximal motor and sensory performances in multiple behavioral tests several weeks after an mild SCI even in the presence of histological abnormalities.

In the present investigation, we developed an appropriate condition to produce a mild SCI in mice. Mice given the mild SCI showed spontaneous functional full recovery from low motor performances in the BMS, rotarod test and beam walking test several weeks after the SCI. The spontaneous functional full recovery was also seen in a sensory test after the SCI. The histological examinations were done using the fully-recovered SCI mice.

## 2 Materials and methods

### 2.1 Mice

Animal care and treatment followed NIH guidelines and were approved by the Animal Resource Committees of Gunma University. All mice were maintained in specific pathogen-free condition in 23 °C, 12 h of light every day, with food and water supplied.

Maximum number of mice in a cage (11 × 19 cm) was 4 mice. Efforts were made to minimize the suffering of mice as much as possible. The number of mice used for the experiments was the minimum necessary to obtain reliable behavioral data (n = 6 or 10 for sham mice and n = 5 to 14 for SCI mice). Mice were randomly divided into experimental groups. Ten to sixteen-week-old male mice (22–28 g) with C57BL/6 background were used for the study.

### 2.2 Spinal cord contusion injury

Mice were anesthetized with ketamine/xylazine (100 mg/kg and 15 mg/kg, respectively, i.p.) before receiving spinal cord injuries. Mice underwent a laminectomy after having their backs shaved. The SCI procedure essentially followed that of Young, 2002 [9]. The exposed spinal cord was contused by a 5.6 g rod with an impact head 1 mm in diameter that was placed 6.25 mm (mild injury), 9 mm (moderate injury) or 12.5 mm (severe injury) above the spinal cord around T10 to allow it to drop using an Impactor model-III spinal cord contusion system (W. M. Keck Center for Collaborative Neuroscience, The State University of New Jersey). After the injury, the wound was sutured using nylon thread. Mice that had cystitis or wound infections were excluded from the analysis.

### 2.3 BMS

BMS is a reliable measure [4] and has been frequently used to evaluate motor functions after SCI in mice. The motor function of the hind limbs was scored using the open-field BMS [4] for 3 min. Briefly, patterns of limb movement such as ankle movement, plantar stepping and paw positions were visually inspected. The score ranges from 0 to 9.

For comparison among mild, moderate and severe SCI, the scoring was done before the injury, and at 3 h, 1 week, 2 weeks and 3 weeks after SCI. For long-term observation after mild SCI, the BMS score was obtained before the injury, 3 hours and 3 days after the injury and every week from 1 to 10 weeks after the injury.

The mice were allowed to walk spontaneously, and their hind limb movements were observed by 3 examiners positioned across the mice from each other to observe both sides of the mice. When the 3 observers had different scores, the final score was determined after discussion. If the score was different between the right and left hind limbs, an averaged value was used.

### 2.4 Rotarod test

Rotarod test was carried out as described [10]. A Rota-Rod treadmill (Muromachi Kikai, Tokyo, Japan) consisted of a plastic rod (3 cm in diameter, 10 cm in length) flanked by two large round plates (57 cm in diameter). Mice were placed on the rod when it was rotating at a constant 4 rpm speed. Then, the rod began to continuously accelerate from 4 to 40 rpm over 5 min. The time each mouse spent on the rod was automatically measured. When a mouse was able to stay on the rod until the cut-off time (5 min), the mouse was removed from the rod, and the maximum time (5 min) was recorded. Testing was completed twice with an interval of 5 min, and the average time for the 2 trials was used for statistical analysis.

### 2.5 Beam walking test

The principle of beam walking test was previously described [7]. The apparatus for the beam walking test consisted of a horizontal round bar 100 cm long and 11 mm in diameter that was elevated 50 cm above a bench; a black box was attached to one end of the bar. The opposite side had a starting point that was 10 cm inside from the end of the beam. Before the SCI, mice were trained to traverse the elevated, narrow beam from the starting point toward the black box 3 times, with intervals of 10 min.

The test was recorded by video camera. The distance each mouse walked on the beam from the starting point toward the goal point, which was located 10 cm before the black box, before dropping from the beam was measured. The cut-off time was set at 120 sec for all mice. Testing was completed twice with an interval of 10 min, and a mean value was calculated from the two experiments for each mouse. The percentage of mice that did not drop from the beam was also quantified.

### 2.6 Dynamic touch test

All mice were acclimated to the testing room and the examiner by letting mice grasp the upper lid of the home cage with their forelimbs when the examiner touches mice gently before behavioral testing. A brush test to examine sensitivity to light touch was carried out as described [11]. Briefly, gentle stroking of hindpaw from heel to toe using a soft paintbrush (12 × 2 mm) was given to mice 10 times with an interval of 30 sec. The sensitivity was estimated by the paw withdrawal frequency from the 10 stimuli. The testing was completed twice with an interval of 1 min, and a mean value was calculated from the two experiments for each mouse.

### 2.7 Histology

Mice were transcardially perfused with 4 % paraformaldehyde in 0.1 M PBS. The entire vertebrae and spinal cord were post-fixed in the same fixative solution overnight at 4 °C. After decalcification, longitudinal and transverse thoracic vertebral blocks containing the T10 lesion center were dissected and embedded in paraffin. Tissue sections 2.5 *μ*m in thickness were prepared from paraffin-embedded blocks using a microtome. After deparaffinization with xylene and treatment with graded alcohol, the sections were processed for Klüver-Barrera staining and HE staining as previously described [12, 13, 14].

Immunofluorescence staining was performed using myelin basic protein (MBP) antibody (Santa Cruz Biotechnology, Dallas, TX) essentially as described [15]. The sections were counterstained with DAPI. The fluorescence signals were obtained using a confocal laser microscope (ZEISS, LSM 880).

### 2.8 Statistical analysis

The mean values with error bars are shown on graphs. The error bar represents standard deviation. Because many data were not normally distributed, we applied non-parametric tests. Statistical analysis of the differences between groups was determined using Kruskal-Wallis test with Steel-Dwass analysis or Mann-Whitney's U test. In addition, Friedman test followed by Scheffe test was also used for repeated measures as previously described [16]. A *p* value less than 0.05 was defined as statistically significant.

## 3 Results

### 3.1 Spontaneous functional full recovery from impaired performances in motor and sensory tasks after a mild SCI

Although a severe SCI likely leads to extensive neuron loss that results in irreversible motor deficits even after a long-term recovery period, a mild SCI might enable full recovery from initial motor deficits. We searched for an experimental procedure to produce mild SCI.

In a previous report, a rod was dropped from a height of 12.5 mm onto the exposed spinal cords of rats to produce a mild SCI using the MASCIS Impactor [17]. Since it was assumed that the height of 12.5 mm would produce a more severe SCI in mice than in rats, the height of 12.5 mm was assumed to produce the most severe injury in mice, whereas 9 mm and 6.25 mm were assumed to create moderate and mild conditions, respectively.

**Fig. 1:**
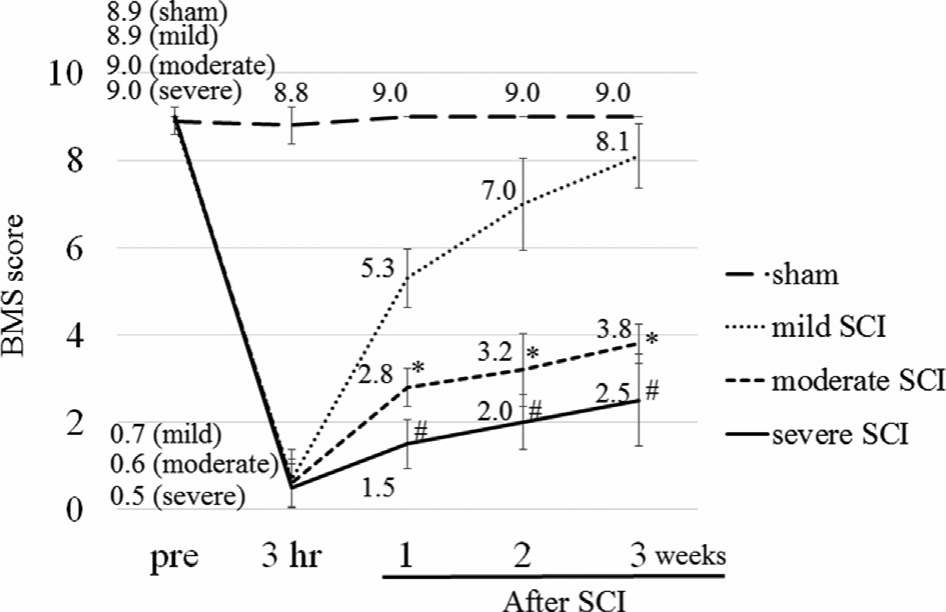
BMS scores of mice after SCI with different severity. The severity was determined by changing the place where the rod was released above the exposed spinal cord at around T10 (6.25 mm, 9 mm and 12.5 mm for mild, moderate and severe injuries, respectively). The BMS score was determined at 3 h, 1 week, 2 weeks and 3 weeks after SCI (n = 10, 5 and 6 for mild, moderate and severe injuries, respectively). Sham-operated mice were also tested (n = 10). The mean values are shown on the graph. The values among mild, moderate and severe SCI were compared. Kruskal-Wallis test with Steel-Dwass analysis, *p < 0.01: mild vs. moderate; #p < 0.01: mild vs. severe.

The BMS score was repeatedly measured at 3 h, 1 week, 2 weeks and 3 weeks after the SCI (Fig. 1). The scores were lowest at 3 h after the SCI and increased over time in all SCI groups, but not in sham group. As expected, the BMS sensitively reflected SCI severity gradations (Fig. 1). When we compared the scores among mild, moderate and severe SCI groups with Kruskal-Wallis test, significant differences were found among the 3 groups at 1 week (p < 0.001), 2 weeks (p < 0.001} and 3 weeks (p < 0.001) after SCI. Steel-Dwass test revealed that the BMS scores of moderate SCI and severe SCI were significantly lower than those of mild SCI at 1 week (both, p < 0.01), 2 weeks (both, p < 0.01) and 3 weeks (both, p < 0.01) after the injury.

**Fig. 2:**
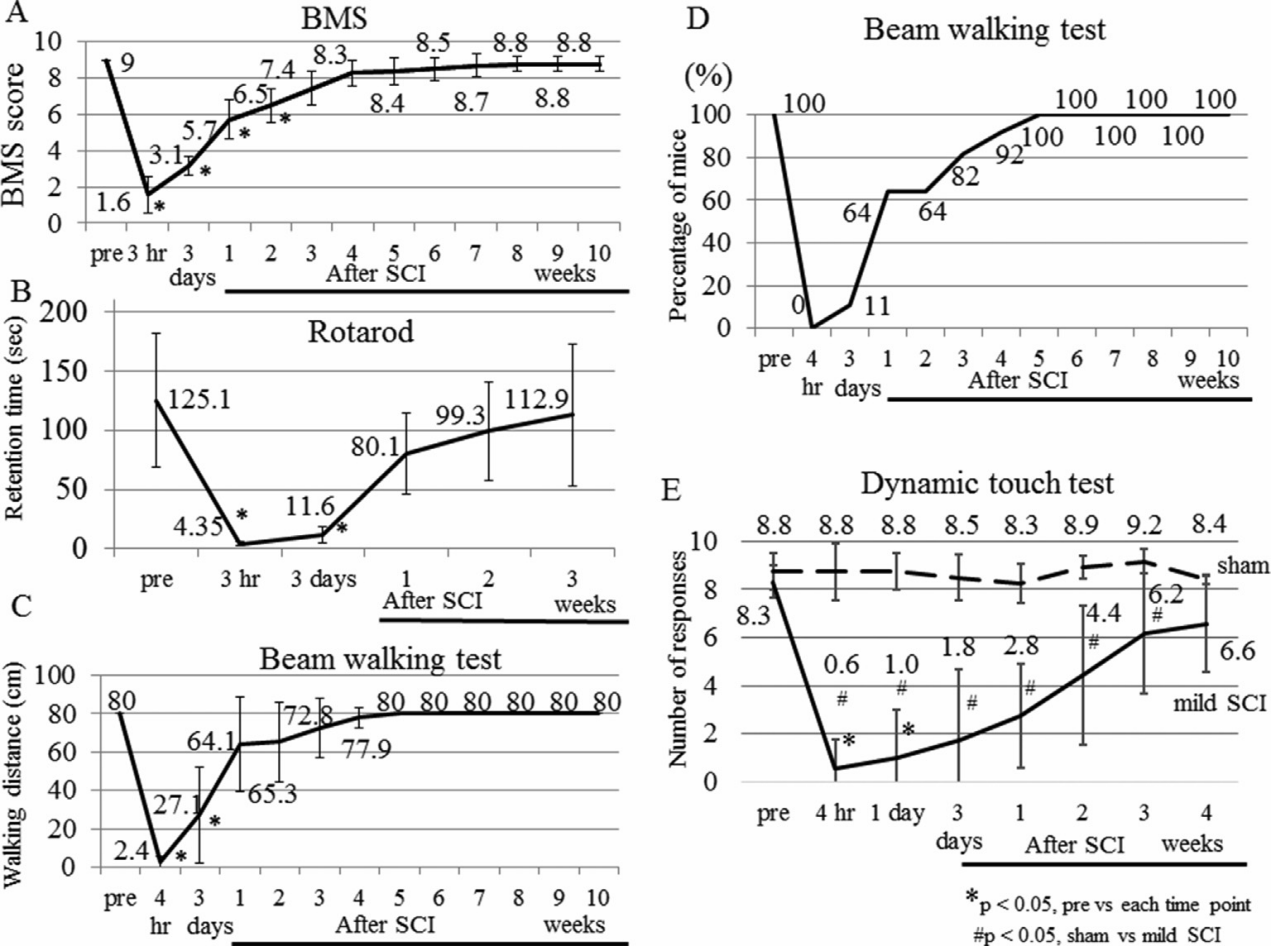
BMS, rotarod test, beam walking test and dynamic touch test performances after mild SCI. (A) Long-term observation of BMS scores. The motor function of the hind limbs was tested using BMS before (pre) the injury, 3 hours and 3 days after the injury and every week from 1 to 10 weeks after the injury (n = 14). (B) Accelerating rotarod test. Average retention time on the rod was measured before the SCI (pre), 3 hours and 3 days after the SCI and every week from 1 to 3 weeks after the SCI (n = 10). (C, D) Performance in the beam walking test was expressed by the distance the mice walked on the beam (C) or by percentage of mice that did not drop from the beam (n = 14) (D). The test was performed before the SCI, 4 hours and 3 days after the SCI and every week from 1 to 10 weeks after the SCI. (E) Dynamic touch test of sham-operated (n = 6) and mild SCI (n = 6) mice at 4 h, 1 day, 3 days, 1 week, 2 weeks, 3 weeks and 4 weeks after SCI. The number of paw withdrawal from 10 stimuli of gentle touch was shown. The mean values are shown on the graphs. The value at each point was compared with that of pre-SCI. Friedman test followed by Scheffe test, *p < 0.05. The values at each time point were also compared between sham and mild SCI groups. Mann-Whitney's U test, #p < 0.05.

Given the determination of the protocol to produce mild SCI, we studied whether the BMS scores at several weeks after the mild SCI are indistinguishable to that at pre-SCI. The BMS score changed over time (Friedman test, p < 0.001). The BMS score before the injury was 9.0 ± 0.0. At 3 days after the mild SCI, the score decreased to 3.1 ± 0.5, which is significantly lower than that before the SCI (Scheffe test, p < 0.001) (Fig. 2A). The significant differences were also found at 1 week (p = 0.002) and 2 weeks (p = 0.02). Then, the score increased to 7.4 ± 0.9 at 3 weeks after the SCI, and the high score was maintained until 10 weeks after the SCI. The scores at 3–10 weeks were not different from that before the SCI (p = 0.21, 0.99, 1.0, 1.0, 1.0, 1.0, 1.0, 1.0, at 3, 4, 5, 6, 7, 8, 9 and 10 weeks, respectively).

We were then motivated to study if the full recovery of motor performances could be seen in other behavioral tests after a mild SCI. Both the rotarod test and beam walking test are well-established behavioral tests that evaluate motor coordination in mice. These tests have been applied to genetically engineered mice to find the physiological role of molecules of interest. In addition, the rotarod test has been used for the assessment of motor function after spinal cord injury [5, 6].

We performed an accelerating rotarod test using mice that had a mild SCI. The retention time changed over time (Friedman test, p < 0.001). The time was approximately 125.1 ± 56.3 sec before the SCI and decreased to 4.4 ± 1.7 sec at 3 h after the SCI, which was significantly shorter than that before the SCI (Scheffe test, p = 0.003) (Fig. 2B). The significant difference was also found at 3 days (p = 0.04). At 1 week, 2 weeks and 3 weeks after the SCI, however, the times were not significantly different from that before the SCI (p = 0.97, 1.0 and 1.0 at 1 week, 2 weeks and 3 weeks, respectively).

The beam walking test has been commonly used to test sensorimotor function after traumatic brain injury and stroke [7]. Performance in the beam walking test was evaluated by the distance the mice walked on the beam before dropping from the beam because the majority of the SCI mice failed to reach the goal point immediately after the SCI. The distance changed over time (Friedman test, p < 0.001). The distance decreased to almost 2.4 ± 3.5 cm immediately after the SCI (Scheffe test, p < 0.001) (Fig. 2C). The significant difference was also found at 3 days compared with pre-SCI (p < 0.001). However, the mice traveled distances at 1–10 weeks after the SCI comparable to that before the SCI (p = 0.81, 0.92, 1.0, 1.0, 1.0, 1.0, 1.0, 1.0, 1.0, 1.0 at 1 week, 2 weeks, 3 weeks, 4 weeks, 5 weeks, 6 weeks, 7 weeks, 8 weeks, 9 weeks and 10 weeks, respectively). Likewise, the percentage of mice that did not drop from the beam for 120 sec was 100 % at 5 weeks after the SCI (Fig. 2D). Collectively, performances in the BMS, rotarod test and beam walking test were completely restored in a short time after the mild SCI.

We also assessed whether mice given the mild SCI recover from a deficit in sensory system in a short time as well. We chose dynamic touch test to assess the sense of light touch because the dorsal spinal cord is responsible for cutaneous sensory modalities [11]. The sensitivity was estimated by the number of paw withdrawal after 10 stimuli.

As a control, disturbed performances in dynamic touch test were not recognized in sham-operated mice (Fig. 2E). In mild SCI group, the number changed over time (Friedman test, p < 0.001). Although significantly fewer numbers of paw withdrawal were recognized in mice at 4 h and 1 day after the mild SCI compared to those before SCI (Scheffe test, p = 0.02 and 0.048 at 4 h and 1 day, respectively), the numbers were not significantly different at 3 days, 1 week, 2 weeks, 3 weeks and 4 weeks post SCI compared to that before SCI (p = 0.15, 0.51, 0.96, 1.0 and 1.0 at 3 days, 1 week, 2 weeks, 3 weeks and 4 weeks, respectively) (Fig. 2E). We then compared the values between sham and mild SCI groups. A marked difference between the groups was found at even 1 week when hindpaw movement was extensively restored (BMS score 5–6). The significant differences were seen until 3 weeks. Then, the value at 4 weeks after mild SCI was not significantly different from that of sham mice (Mann-Whitney's U test, p = 0.16) (Fig. 2E). Collectively, the functional full recovery of the sense of light touch was also seen after a mild SCI.

### 3.2 Histological examination of mild SCI mice

**Fig. 3:**
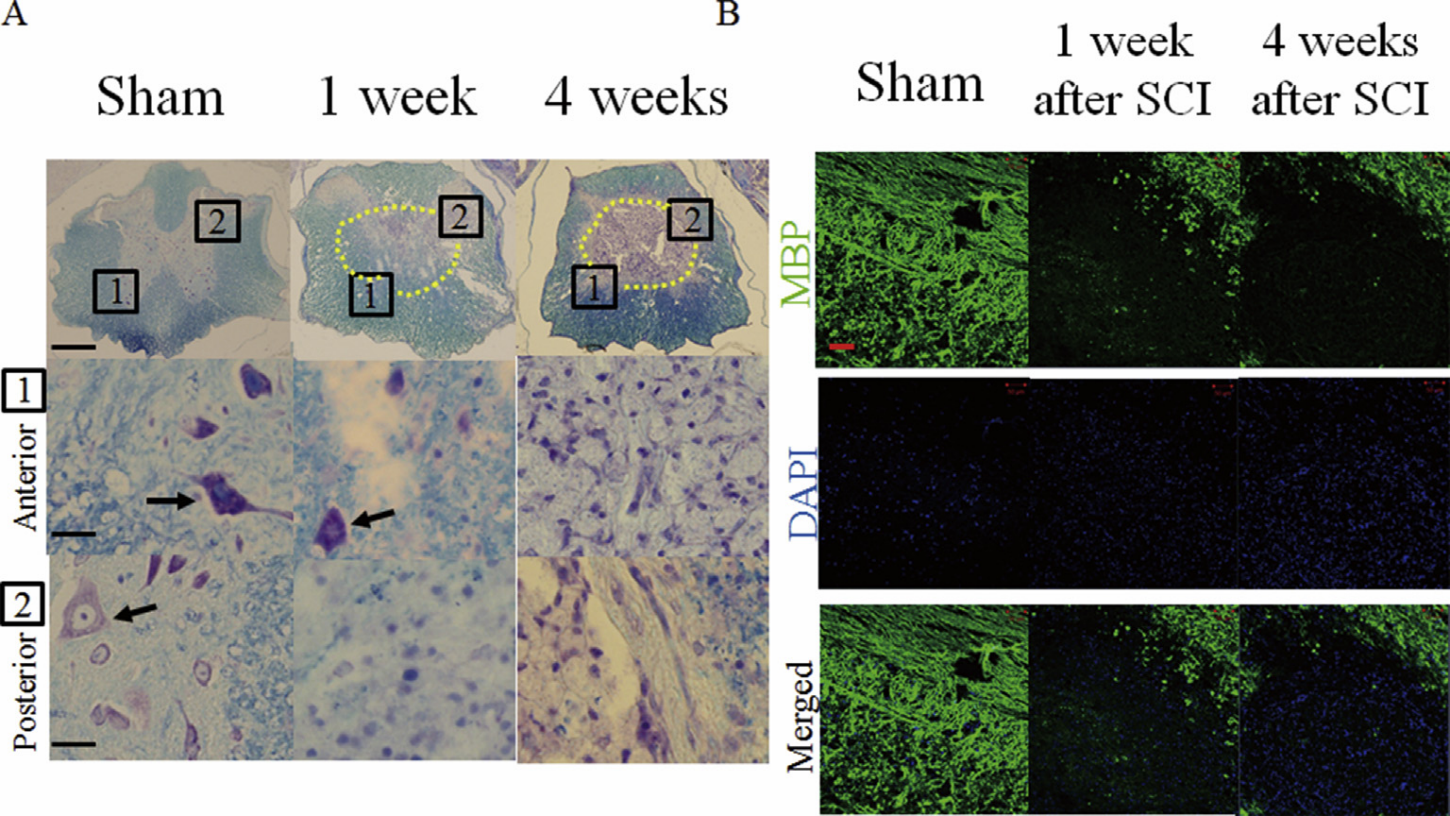
Loss of myelin in the spinal cord in mice at 4 weeks after mild SCI. (A) Klüver-Barrera staining was performed using transverse sections from sham-operated mice, mice at 1 week and 4 weeks after mild SCI. The lesion area in the low magnifications images are surrounded by the dotted lines. The boxes in the low magnification images (top) located in the anterior (middle) and posterior (bottom) spinal regions are magnified. Scale bars, 300, 20 and 20 *μ*m in top, middle and bottom, respectively. Arrows indicate soma of neurons. Sections from 3 mice for each group were stained. (B) Immunofluorescence staining of longitudinal sections of the spinal cord from sham-operated mice and mice at 1 week and 4 weeks after mild SCI with MBP antibody (green). The sections were also stained with DAPI (blue). Sections from 3 mice for each group were stained. Scale bars, 50 *μ*m.

We then examined whether the fully recovered SCI mice had anatomical defects or not. Klüver-Barrera staining was performed using transverse spinal sections of mice 1 week and 4 weeks after a mild SCI as well as sham-operated mice. Numerous neurons in the anterior and posterior spinal regions were found in sham-operated mice. However, much fewer number of neurons were found at 1 week and 4 weeks after mild SCI (Fig. 3A). The density of myelin was obviously decreased in the mice at 1 week and 4 weeks after mild SCI compared to sham-operated mice (Fig. 3A).

We confirmed the extensive loss of myelin of SCI mice using immunostaining with anti- MBP antibody. Strong MBP signals were found in the spinal cord of sham-operated mice (Fig. 3B). However, the strong MBP signals were discontinued at the lesion site of the spinal cord in mice at 1 week after a mild SCI (Fig. 3B). Remyelination spontaneously occurs in several animal models after SCI [18]. Remyelination begins to appear at 2–4 weeks and is completed within 3 months after an SCI in rodents [19]. However, the loss of myelin was still definitely observed at 4 weeks post-mild SCI (Fig. 3B). DAPI staining revealed accumulation of presumable inflammatory cells in the injured sites in sections post SCI (Fig. 3B).

**Fig. 4:**
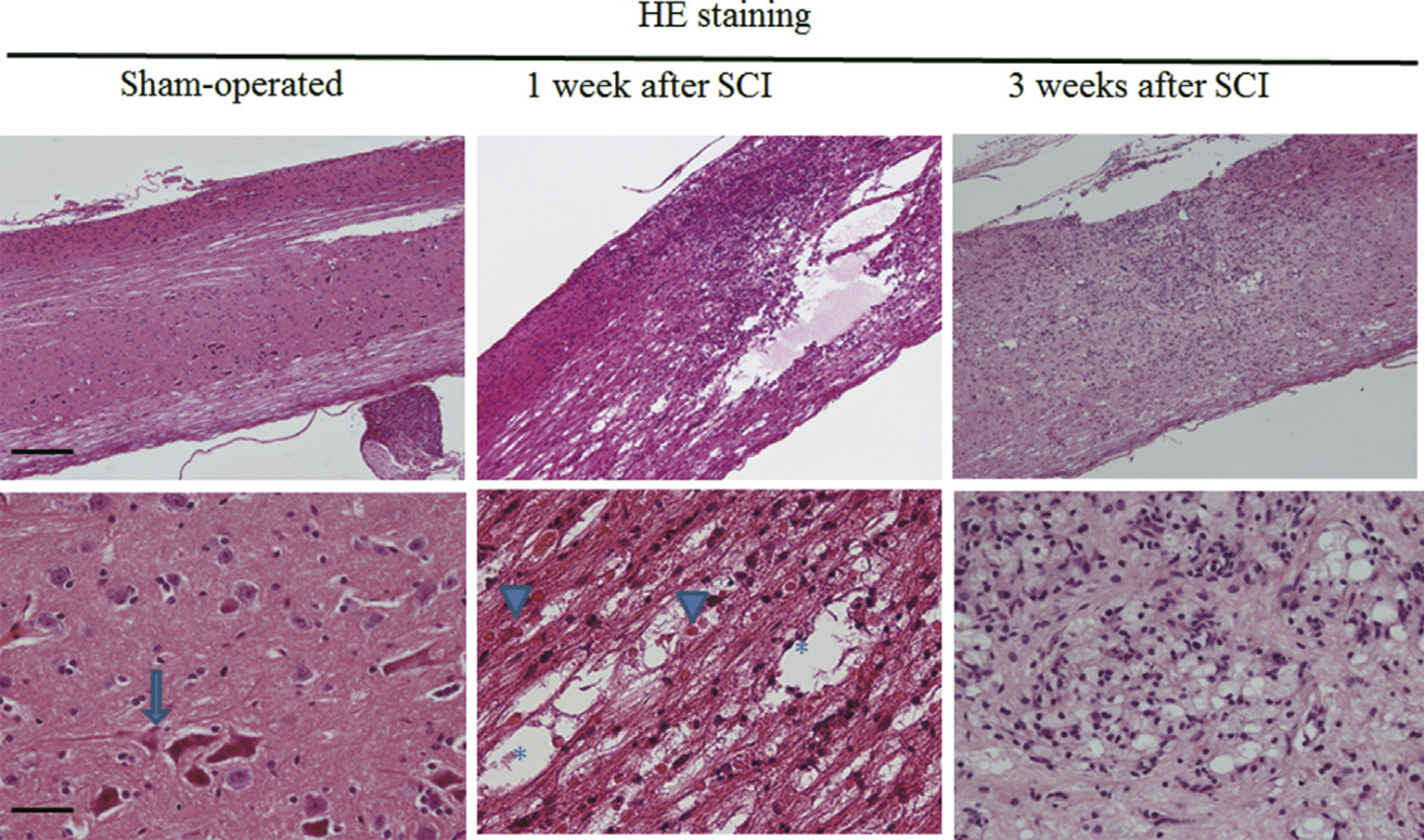
Cavity and bleeding at 1 week and 3 weeks after mild SCI. Low (top) and high (bottom) magnification images of HE-stained longitudinal sections of the spinal cord from sham-operated mice and mice at 1 week and 3 weeks after a mild SCI. An arrow, arrowheads and asterisks indicate a neuron, red blood cells and cavities, respectively. Sections from 4 mice for each group were stained. Scale bars, 200 *μ*m and 50 *μ*m in top and bottom panels, respectively.

We finally carried out HE staining (Fig. 4). Again, the sham operation did not injure the spinal nerve. At 1 week after the mild SCI, red blood cells, which reflect bleeding, were frequently observed in the lesion area, and cavities were also found (Fig. 4). In addition, the infiltration of mononuclear cells was seen. The size of cavities seemed to be smaller and degree of bleeding tended to be weaker at 3 weeks than those at 1 week after the SCI (Fig. 4).

## 4 Discussion

In the present investigation, we have shown that mice with mild SCI were completely recovered from impaired motor functions and low sensitivity to light touch in a short time. Nevertheless, the fully-recovered SCI mice still exhibited loss of myelin in the spinal cord. Neonatal animals generally have more plasticity in the nervous system after injuries than the adult. Regarding cases of SCIs, the complete functional recovery of voluntary hindlimb movements was rapidly accomplished in neonatal mice [20]. In contrast, adult animals often display spontaneous partial recovery of motor functions after SCI. However, some past studies have shown full recovery in a single motor behavioral test after SCI or cortical impact in rodents. For example, BBB scores of rats at 60 days post-mild SCI almost recovered to a normal level (BBB score = 19) [5]. The full recovery of accelerating rotarod performance was also observed in mice given a cortical impact. The rotarod performance of the mice that underwent the mild impact was lower than those of the sham mice at 1, 2 and 3 days post-surgery. Then, their performance returned to the same level as that of sham mice at 7–21 days post-surgery [21]. Remarkably, the current study revealed that the motor performances in 3 behavioral tests were fully restored in a short time after SCI. Furthermore, the SCI mice also showed full recovery from low sensitivity to light touch using dynamic touch test.

Concerning the long-term prognosis of SCI patients who have been surgically treated or undergone conservative management, only a few people exhibited complete motor improvement [22]. However, it is practically difficult to obtain information on the prognoses of SCI patients without surgical or conservative treatments. Thus, only animal studies provide us with information on the spontaneous recovery of motor functions after SCI. Although humans and rodents should exhibit different degrees of the spontaneous recovery of motor functions, the complete motor recovery in mice using three tests in the present study reveals the high potential for adaptive changes in the spinal cord after SCI.

Regarding the severity of the injury, SCIs are divided into complete and incomplete SCIs in humans. Incomplete SCIs are more common than complete SCIs [1]. Mild SCIs should have better prognoses than severe SCIs because initial ASIA scores in SCI patients have proven to be the most important factor for prognosticating motor improvements [22]. Therefore, the complete recovery of the motor behaviors after the mild SCI in mice in this study is noteworthy. On the other hand, multiple sclerosis in humans, an another type of demyelination, leads to significant motor deficits even in the absence of extensive injury such as traumatic injury. Possibilities remain that the three tests could not detect the motor deficits of the SCI mice and other tests might detect the deficits in motor performances.

There are multiple possibilities regarding the mechanisms that enable functional full recovery. Structural adaptation of neurons after SCI is extensively seen in neonatal animals. In neonatal SCI mice showing approximately 50 % tissue loss together with a marked reduction in the number of neurons, a compensatory increase in serotonergic innervation and the restoration of the synaptic terminals of motoneurons were observed [23]. Even in the adult, collateral sprouting to the injury site [24, 25, 26] from preserved regions in the spinal cord might aid recovery. In this study, the degrees of cavities and bleeding seemed to be attenuated after mild SCI. Since impairments in microcirculation in the spinal cord lead to the dysfunction of neurons and glial cells [27], the degrees of cavities and bleeding in SCI mice might be related to behavioral recovery. If the specific factors that control the full spontaneous motor recovery in mice would be identified in future, the finding likely contributes to therapeutic research for SCI.

## Declarations

### Author contribution statement

Yohei Kakuta: Performed the experiments; Analyzed and interpreted the data; Wrote the paper.

Anna Adachi, Marino Yokohama, Toshiki Horii, Tokue Mieda: Performed the experiments; Analyzed and interpreted the data.

Yoichi Iizuka, Haku Iizuka: Conceived and designed the experiments; Analyzed and interpreted the data.

Kenji Takagishi: Conceived and designed the experiments; Analyzed and interpreted the data; Contributed reagents, materials, analysis tools or data.

Hirotaka Chikuda: Analyzed and interpreted the data; Wrote the paper.

Kazuhiro Nakamura: Conceived and designed the experiments; Performed the experiments; Analyzed and interpreted the data; Contributed reagents, materials, analysis tools or data; Wrote the paper.

### Funding statement

This research did not receive any specific grant from funding agencies in the public, commercial, or not-for-profit sectors.

### Competing interest statement

The authors declare no conflict of interest.

### Additional information

No additional information is available for this paper.
